# Human Schlafen 5 regulates reversible epithelial and mesenchymal transitions in breast cancer by suppression of ZEB1 transcription

**DOI:** 10.1038/s41416-020-0873-z

**Published:** 2020-06-03

**Authors:** Guoqing Wan, Jiang Zhu, Xuefeng Gu, Yue Yang, Yihao Liu, Zhizheng Wang, Yuxia Zhao, Hailong Wu, Gang Huang, Changlian Lu

**Affiliations:** 1 Shanghai Key Laboratory of Molecular Imaging, Shanghai University of Medicine and Health Sciences, Shanghai, China; 2 Collaborative Research Center, Shanghai University of Medicine and Health Sciences, Shanghai, China; 3 Department of Orthopedics, the First Affiliated Hospital of Harbin Medical University, Harbin, Heilongjiang China; 4 Department of Pathology, Mudanjiang Medical University, Mudanjiang, China; 5 Key Laboratory of Human Disease Comparative Medicine, Ministry of Health, Institute of Laboratory Animal Science, Chinese Academy of Medical Sciences & Comparative Medical Center, Peking Union Medical College, Beijing, China

**Keywords:** Epithelial-mesenchymal transition, Breast cancer

## Abstract

**Background:**

Human Schlafen 5 (SLFN5) has been reported to inhibit or promote cell invasion in tumours depending on their origin. However, its role in breast cancer (BRCA) is undetermined.

**Methods:**

Differential expression analyses using The Cancer Genome Atlas (TCGA) data, clinical samples and cell lines were performed. Lentiviral knockdown and overexpression experiments were performed to detect changes in cell morphology, molecular markers and invasion. Chromatin immunoprecipitation-sequencing (ChIP-Seq) and luciferase reporter assays were performed to detect the SLFN5-binding motif.

**Results:**

TCGA, clinical samples and cell lines showed that SLFN5 expression was negatively correlated with BRCA metastasis. SLFN5 knockdown induced epithelial–mesenchymal transition (EMT) and enhanced invasion in BRCA cell lines. However, overexpression triggered mesenchymal–epithelial transition (MET). SLFN5 inhibited the expression of ZEB1 but not ZEB2, SNAI1, SNAI2, TWIST1 or TWIST2. Knockdown and overexpression of ZEB1 indicated that it was a mediator of the SLFN5-governed phenotype and invasion changes. Moreover, SLFN5 inhibited *ZEB1* transcription by directly binding to the SLFN5-binding motif on the *ZEB1* promoter, but a SLFN5 C-terminal deletion mutant did not.

**Conclusion:**

SLFN5 regulates reversible epithelial and mesenchymal transitions, and inhibits BRCA metastasis by suppression of *ZEB1* transcription, suggesting that SLFN5 could be a potential target for BRCA therapy.

## Background

The mouse Slfn family has been implicated in various physiological or pathological processes, including T-cell activation, thymocyte maturation, fibroblast and tumour cell proliferation.^1–4^ However, the human SLFN family has not been extensively studied, with the exception of SLFN11, which is capable of suppressing HIV replication, and positively correlates with the effect of topoisomerase inhibitors on human cancer cells.^5–7^ There are very few studies on human SLFN5, and the results are inconsistent. For example, human SLFN5 is found at a low level in melanoma and renal cell carcinoma, and plays inhibitory roles in tumour invasion.^8,9^ However, SLFN5 is positively correlated with malignant progression in glioblastoma, where it acts as a co-repressor with signal transducer and activator of transcription 1 (STAT1) in interferon-mediated responses.^10^ We previously found that SLFN5 inhibited invasion by repressing membrane- tethered-1 matrix metalloproteinase (MT1-MMP) expression in five cell lines from lung cancer, colorectal cancer, fibrosarcoma, renal clear-cell cancer and breast cancer.^11^ To date, the function of human SLFN5 is still largely unknown.

EMT is a key driver of cancer metastasis; it is characterised by the loss of epithelial morphology due to the loss of epithelial intercellular junctions such as E-cadherin, and the gain of mesenchymal morphology along with the upregulation of mesenchymal molecules such as vimentin and N-cadherin.^12–15^ Several key transcription factors, such as SNAI1, SNAI2, ZEB1, ZEB2, TWIST1 and TWIST2, have been found to contribute to EMT by either suppressing E-cadherin expression or inducing the expression of pro-metastasis genes.^12–31^ Among them, ZEB1 is a primary contributor,^32^ and it is aberrantly overexpressed in many types of cancers, including BRCA, where it promotes cancerous metastasis.^33–36^ Many extracellular and intracellular factors have been identified in the regulation of ZEB1 expression, and their underlying mechanisms have also been explored to some extent.^20–23^ However, whether there is a relationship between SLFN5 and ZEB1 is unexplored.

BRCA is the most common malignancy that poses a serious threat to women’s health, and metastasis is the leading cause of death for patients with BRCA.^37^ In the present study, we investigated the role of SLFN5 in BRCA progression using bioinformatic tools, gain or loss of expression, ChIP-Seq analyses and *ZEB1* promoter activity assays, and we clearly demonstrated that human SLFN5 is a breast cancer suppressor, and that its anti-oncogenic functions are realised through a direct modulation of ZEB1 expression in breast cancer.

## Methods

### Cell culture

The human BRCA cell lines MCF7, MDA-MB-231, T47D and BT549 were purchased from the American Type Culture Collection (ATCC, Manassas, Virginia). MDA-MB-453, BT-474 and ZR-75-1 were purchased from Beijing Beina Chuanglian Biotechnology Research Institute (Beijing). All cell lines were routinely cultured in high-glucose Dulbecco’s modified Eagle’s medium (DMEM, HyClone) that was supplemented with 10% foetal bovine serum (Gibco, Paisley, Scotland), 100 U/mL penicillin, 100 μg/mL streptomycin (HyClone) and 2 mM L-glutamine (HyClone). Cells were maintained in an incubator containing 5% CO_2_.

### TCGA BRCA data analysis

We downloaded a processed TCGA breast cancer fragments per kilobase of transcript per million mapped reads (FPKM) gene expression matrix from the UCSC Xena platform (https://gdc.xenahubs.net), and we acquired the clinical information for the corresponding samples from TCGA (https://cancergenome.nih.gov). BRCA patients were ranked according to TNM stage, primary cancer (4 > T > 0, N = 0, M = 0), lymphatic metastasis (N > 0, M = 0) and distant metastasis (M > 0). In addition, oestrogen receptor (ER), progesterone receptor (PR) and human epidermal growth factor receptor-2 (HER2) clinical information was used to divide patients into luminal A (ER^+^ and/or PR^+^, HER2^−^), luminal B (ER^+^ and/or PR^+^, HER2^+^), HER2^+^ (ER^−^, PR^−^, HER2^+^) and basal-like/triple-negative (ER^−^, PR^−^, HER2^−^) groups.^38^

### Immunostaining and H&E staining of BRCA tissues

The patients consented for their tissue samples from BRCA resection operations to be used for the present study, and the study was approved by the Ethics Committee at the Shanghai University of Medicine and Health Sciences. BRCA samples were divided into primary unmetastasised group (*n* = 5) and distant metastasised group (*n* = 5) for each molecular subtype, with adjacent normal breast tissues as control. BRCA tissues were fixed in 4% paraformaldehyde and then were embedded in paraffin. Four-micrometre sections containing both tumour and adjacent normal breast tissues as normal controls were processed and immunostained with an anti-SLFN5 antibody (Sigma-Aldrich) or an anti-ZEB1 antibody (Cell Signaling Technology), which was followed by secondary antibody incubation. H&E staining was performed to identify cancer pathological features. Expression scores of SLFN5 and ZEB1 were calculated based on the percentage of positive cells and staining intensity, using the following computational formula: the total score = 10 × positive cells% × intensity value. The intensity value ranged from 0 to 1.0.

### Human SLFN5 and ZEB1 overexpression and shRNA constructs

Human SLFN5 (NM_44975.3) and ZEB1 (NM_001128128.2) full-length coding sequences were cloned into lentiviral vectors GV358 and pLVX-IRES-NEO, respectively, and each empty vector served as a transfection control. Human SLFN5 shRNA (5′-GACUCAGACUCCAACGAAUTT-3′) and ZEB1 shRNA (5′-CCGGTGTCTCCCATAAGTATCAATTCTCG-3′) were introduced into the lentiviral vector stem-loop backbone, and a scrambled shRNA for each was constructed as a negative control. Each of these constructs was transfected together with packaging vectors (psPAX2 and pMD2G) into HEK293T cells. Forty-eight hours after transfection, supernatants were collected, and lentiviral particles were concentrated. Lentiviral particles containing SLFN5 and the control were transfected into MDA-MB-231 and BT549 cells, and lentiviral particles containing SLFN5 shRNA and control shRNA were transfected into MCF7 and T47D cells. GFP-positive cells were sorted using flow cytometry, and then were cultured in puromycin at a concentration of 0.25–0.5 μg/ml to maintain stably transfected cell lines. All stably transfected cell lines were verified by detection of GFP fluorescence under an inverted fluorescence microscope, and by detection of flag or SLFN5 protein expression using western blotting (WB) analysis. In some indicated experiments, lentiviral particles containing ZEB1 were transfected into MDA-MB-231 cell lines stably expressing SLFN5, and lentiviral particles containing ZEB1 shRNA were transfected into MCF7 cell lines stably silencing SLFN5.

### Analysis of *ZEB1* promoter activity by dual-luciferase reporter gene systems

The *ZEB1* promoter and the indicated length upstream from the transcription starting site (TSS) was amplified from the HEK293T genome by PCR, and then was ligated into the pGL-3 basic vector (Promega) with the cloning sites *Mlu*I/*Xho*I; the recombinant vectors were named ZEB1–1–5. The primers used for vector cloning are listed in Supplementary Table S1. The intact wild-type sequence, 5′-TCCCCTCA-3′ (–487 to –494 upstream from TSS) of the ZEB1–5, was mutated into 5′-CTTTTCTG-3′, producing a construct named mutated binding site #1. All constructs were verified by sequencing. Constructs were transfected into MCF7 cells with SLFN5 knocked down or MDA-MB-231 cells overexpressing wild-type SLFN5 or C-terminal deletion mutant (SLFN5delC; C-terminal 335 amino acids were deleted). The SLFN5delC mutant was constructed using N-terminal 1608 nucleotides (encoding N-terminal 536 amino acids) of the SLFN5 coding sequences, which were transfected into MDA-MB-231 cells for luciferase activity assays. Firefly and Renilla luciferase activities were detected using the Dual-Luciferase Reporter Assay System according to the manufacturer’s instructions (Promega).

### Real-time PCR analysis

The total RNA was isolated from cancer cells and reverse-transcribed to produce cDNA using a Golden 1st cDNA synthesis kit containing gDNA Remover with oligo (dT) primers according to the manufacturer’s instructions (Haiji, China). Real-time PCR was performed using SYBR Green master mix (Applied Biosystems) on a real-time PCR detection system (ABI 7500). Relative quantification of mRNA levels was calculated as fold change using the standard method, 2^−∆∆CT^ = (CT_target_ – CT_β-actin_)_sample_ – (CT_target_ – CT_β-actin_)_control_. In some indicated experiments, traditional PCR followed by agarose gel electrophoresis was performed to identify the size of amplified fragments. The primers used are listed in Supplementary Table S1.

### WB analysis

Protein samples extracted from cancer cells were subjected to SDS-PAGE followed by transfer onto nitrocellulose membranes for primary antibody hybridisation. An anti-SLFN5 rabbit antibody was purchased from Sigma-Aldrich. The following antibodies were purchased from Cell Signaling Technology (Danvers, MA, USA): E-cadherin, Vimentin, N-Cadherin, Zonula occludens protein-1 (ZO-1), SNAI1 (C15D3), SNAI2 and ZEB1. A ZEB2 antibody was purchased from Santa Cruz Biotechnology (Dallas, TX, USA), TWIST1 and TWIST2 antibodies were purchased from Abcam (Cambridge, MA, USA) and HRP-conjugated secondary antibodies were purchased from Beyotime Biotechnology (Shanghai, China). Protein bands hybridised with antibodies on membranes were detected using ultrasensitive ECL chemiluminescence reagent (Beyotime Biotechnology). Band intensity was quantified using Quantity One software (Bio-Rad), and is presented as the mean ± SD of three independent experiments.

### Immunofluorescence cytochemistry analysis

Cells cultured on Lab-Tek II Chambered Coverglass (Thermo Fisher Scientific) were fixed with 1% polyformaldehyde for 2 min, and then were permeabilised by treatment with 0.1% Triton X-100 for 10 min. Cells were incubated with the indicated primary antibodies, which was followed by incubation with Alexa Fluor 594 anti-rabbit or anti-mouse IgG. Nuclei were counterstained with 4′,6′-diamidino-2-phenylindole (DAPI). Images were observed under an inverted fluorescence microscope (Olympus IX71, Tokyo, Japan) or photographed under an inverted laser confocal fluorescence microscope (Leica, Germany). Microscope settings and laser intensity were maintained at unchanged levels throughout the different experimental conditions.

### Migration and invasion in vitro and chick embryo chorioallantoic membrane (CAM) invasion in vivo

In vitro migration experiments were performed using Boyden chambers containing Transwell membrane filter inserts (Corning Costar, USA). For in vitro invasion experiments, Matrigel (BD, USA) was coated on a Transwell membrane filter to enable analysis of cell invasion. The numbers of migrating or invading cells were counted under a light microscope from five fields in a single chamber of three samples (mean ± SD). An in vivo chick CAM invasion assay was performed as reported previously.^39,40^ Briefly, cancer cells were incubated with fluoresbrite carboxylate microspheres (Polysciences, PA, USA) and then were seeded atop CAM for 3 days. CAM samples were fixed in 4% paraformaldehyde, and then were cryosectioned. Images were captured by fluorescence microscopy. The number of cells penetrating the CAM and infiltrating substratum matrix tissues was calculated, and the infiltration depth was measured (mean ± SD, *n* ≥ 3).

### ChIP-Seq analysis

Protein–DNA complexes in MCF7 cells were cross-linked with 1% paraformaldehyde, and then the cells were collected. Nuclei were isolated following treatment with lysis buffer, and DNA was collected following fragmentation by ultrasonic treatment. IP was performed using magnetic protein A/G beads coupled to an anti-SLFN5 antibody, and mouse IgG was used as an antibody- negative control. Immunoprecipitated DNA was released from protein–DNA complexes by digestion with proteinase K, and then it was divided into two parts: one part was used for preparation of the transcriptome-sequencing (RNA-seq) library, and the other was used for real-time PCR identification. The libraries were generated using Illumina TruSeq technology (Illumina, San Diego, CA) and then were sequenced using an Illumina HiSeq 2000 sequencer. The RNA sequencing data were converted into base sequences using off-line Basecaller software (OLB V1.8.0), and the original reads (tags) of a specific length were obtained by filtering these base sequences with Solexa CHASTITY. Next, the DNA sequencing raw reads were preprocessed by filtering out sequencing adapters, short-fragment reads and other low-quality reads. Bowtie (version 0.12.8) was then used to map the clean reads to the human hg19 reference genome. Peak detection was performed by MACS with a minimum *P*-value cut-off of 0.00001 (version 1.4.2, https://pypi.python.org/pypi/MACS/1.4.2). The peak recognition, overlap, subtraction, merge and feature annotation analysis of enriched regions were performed by using the Hypergeometric Optimization of Motif EnRichment 2 (HOMER2) suite (version 3.0, http://homer.ucsd.edu/homer/).^41^ Motif-density histograms were created using HOMER for target regions and promoters defined as −2 kb to +1 b relative to the TSS. The ZEB1 promoter was identified by real-time PCR with the specific primers listed in Supplementary Table S1.

### Statistical analysis

GraphPad Prism 6 software was used to generate graphs and perform statistical analysis (one-way ANOVA test with Tukey’s post hoc test for multiple comparisons).

## Results

### TCGA, clinical samples and cell lines of human BRCA show that SLFN5 is negatively correlated with BRCA metastasis

**Fig. 1:**
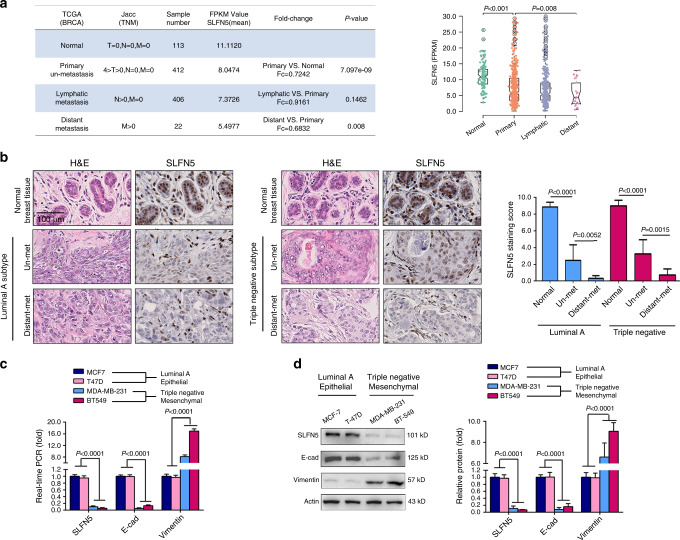
Analyses of human SLFN5 expression in BRCA using TCGA data, clinical samples and cell lines. **a** The processed TCGA breast cancer FPKM gene expression matrix was downloaded from the UCSC Xena platform (https://gdc.xenahubs.net), and clinical information for the corresponding samples was downloaded from TCGA (https://cancergenome.nih.gov). BRCA patients were divided into three groups: primary unmetastasised BRCA, lymphatic metastasised BRCA and distant metastasised BRCA. Differential expression analysis of SLFN5 RNA levels in normal controls and in local primary tumour tissues for each BRCA group. **b** Immunohistochemistry analysis of SLFN5 protein levels in adjacent normal breast tissues, local primary tumour tissues with distant metastasis (Distant-met) or without distant metastasis (un-met) of BRCA luminal A and triple-negative subtypes. SLFN5 expression scores were calculated based on the percentage of positive cells and the staining intensity (mean ± SD, *n* = 5). H&E staining was performed to identify pathological features of cancer. Scale bar: 100 μm. **c** WB analysis of SLFN5 expression in breast cancer cell lines. E-cadherin (E-cad) was used as an epithelial marker, vimentin was used as a mesenchymal marker and actin was used as a loading control. Protein band intensity was quantified using Quantity One software (Bio-Rad), and it is presented as the mean ± SD of three independent experiments. **d** Real-time PCR analysis of the mRNA levels of SLFN5, E-cadherin and vimentin in breast cancer cell lines.

To investigate the role of SLFN5 in BRCA, we first downloaded the processed TCGA breast cancer FPKM gene expression matrix and the clinical information for all 840 BRCA patients and 113 normal individuals. BRCA cases were divided into three groups: primary unmetastasised BRCA (*n* = 412), lymphatic metastasised BRCA (*n* = 406) and distant metastasised BRCA (*n* = 22, Fig. 1a). Differential expression analysis was performed to determine the SLFN5 RNA levels in normal controls and in local primary tumour tissues for each BRCA group. Compared with normal controls, SLFN5 levels were significantly lower in BRCA tumour tissues (Fig. 1a, *P* < 0.001). Moreover, SLFN5 RNA expression in tumour tissues decreased with metastasis (Fig. 1a, *P* = 0.008). We also analysed TCGA data according to molecular subtypes, i.e., luminal A, luminal B, HER2^+^ and triple negative. SLFN5 RNA expression in all subtypes was decreased compared with that of normal tissues. Regarding distant metastasis, the samples of each subtype were too small for statistical analysis (luminal B and triple negative two samples, HER2^+^ only one sample) (Supplementary Fig. S1).

Further, clinical surgical samples of local primary BRCA were collected, and SLFN5 protein levels were examined using immunostaining method. Compared with adjacent normal breast tissues, SLFN5 protein levels were significantly decreased in primary unmetastasised BRCA tumour tissues of each subtype (Un-met) and further decreased in local primary BRCA samples with distant metastasis (Distant-met) compared with Un-met (Fig. 1b, luminal A and triple negative; Supplementary Fig. S2, luminal B and HER2^+^).

In addition, SLFN5 expression was further examined in BRCA cell lines for each subtype with different morphologies and metastasis capacities: MCF7 and T47D (luminal A, unmetastasis), MDA-MB-231 and BT549 (triple negative, high metastasis), MDA-MB-453 (HER2^+^, high metastasis), BT-474 (luminal B, unmetastasis) and ZR-75-1 (luminal B, high metastasis). Consistent with our findings in TCGA and clinical BRCA samples, both WB and real-time PCR assays showed that unmetastasised BRCA cell lines present high levels of both the epithelial marker E-cadherin (E-cad) and SLFN5, while highly metastasised BRCA cell lines present low levels of both, which were further verified by the tendency of vimentin, a mesenchymal marker, to be highly expressed in these highly metastasised BRCA cells (Fig. 1c, d, luminal A and triple-negative cell lines; Supplementary Fig. S3, luminal B and HER2^+^). Taken together, these results suggest that SLFN5 expression could be negatively correlated with the metastatic capacity of BRCA.

### Knockdown of SLFN5 induces EMT in epithelial-like BRCA cells

**Fig. 2:**
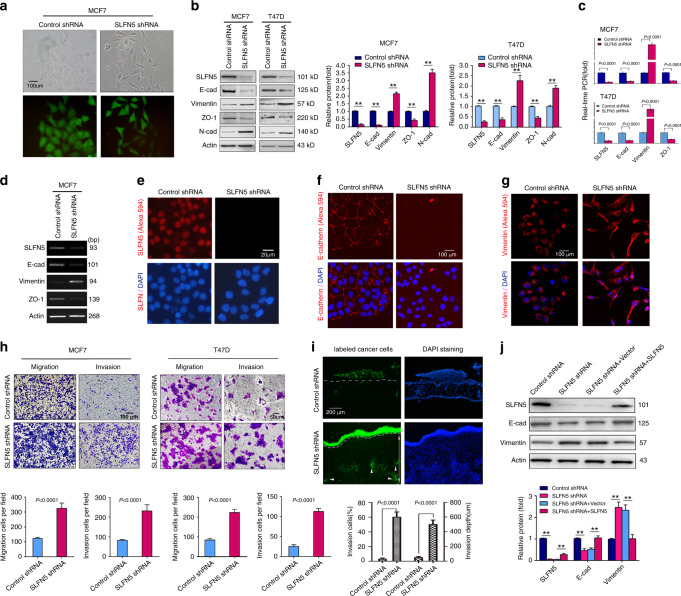
Knockdown of SLFN5 induces EMT in epithelial BRCA cell lines and promotes invasion in vitro and in vivo. **a** Contrast-phase and fluorescent images of MCF7 cell lines stably transfected with lentiviral control shRNA or lentiviral SLFN5 shRNA. Scale bar: 20 μm. **b** WB analysis of the expression levels of SLFN5, epithelial markers (E-cad and Zonula occludens protein-1 (ZO-1)) and mesenchymal markers (vimentin and N-cadherin (N-cad)) in MCF7 and T47D cell lines stably transfected with control shRNA or SLFN5 shRNA. Protein band intensity is quantified and presented as the mean ± SD of three independent experiments. **P < 0.01. **c** Real-time PCR analysis of gene expression in MCF7 and T47D cell lines stably transfected with control shRNA or SLFN5 shRNA. **d** Traditional PCR followed by agarose gel electrophoresis was performed to identify the size of amplified fragments. **e**–**g** Immunofluorescence analyses of SLFN5, E-cadherin and vimentin expression in stably transfected control shRNA or SLFN5 shRNA MCF7 cell lines, stained with the indicated primary antibodies and Alexa Fluor 594-conjugated secondary antibodies. The results were observed with laser confocal fluorescence microscopy. DAPI staining revealed the nuclei. **h** Migration and invasion assays of MCF7 and T47D cell lines stably transfected with control shRNA or SLFN5 shRNA. Migration and invasion cells per field were calculated. **i** Chick CAM invasion analysis of MCF7 cell lines stably transfected with control shRNA or SLFN5 shRNA. The white dotted lines indicate the position of the CAM. The figures shown are representative results of three similar experiments. The white arrowheads indicate cells invading into the chick embryos. The double-headed arrow indicates the depth of cell invasion. DAPI staining of the cell nuclei of both human cancer and chick cells is shown. Cell numbers and depth were quantified as the mean ± SD of three independent experiments. **j** Rescue experiments were performed using human SLFN5 viral particles (because mouse Slfn5 has only 59% homology to humans). The results show that SLFN5 transfection partially rescued SLFN5 knockdown-induced E-cadherin and vimentin changes. Protein band intensity is quantified and is presented as the mean ± SD of three independent experiments. **P < 0.01.

To investigate the functional implications of SLFN5 in BRCA, MCF7 and T47D cells were infected with a lentivirus encoding shRNA against SLFN5. Compared with cells infected with a control shRNA, which showed cobblestone epithelial morphology (Fig. 2a), cells infected with SLFN5 shRNA displayed a scattered spindle-shaped mesenchymal morphology (Fig. 2a). In line with the morphological changes, SLFN5 knockdown led to a decrease in the epithelial markers E-cad and ZO-1, and an increase in the mesenchymal markers vimentin and N-cadherin (N-cad) (Fig. 2b–d, *P* < 0.01 or *P* < 0.001). Meanwhile, immunofluorescent staining (IF) also confirmed the decrease in E-cad and the increase in vimentin in cells with SLFN5 knocked down (Fig. 2e–g). Consistent with previous studies showing the nuclear localisation of SLFN5,^9,11^ our IF assays also showed that SLFN5 is exclusively located in the nucleus (Fig. 2e). To investigate whether SLFN5 knockdown can enhance EMT functionally, in vitro Transwell migration and invasion assays were performed with non-invasive MCF7 and T47D cells. Compared with the shRNA control, cells with SLFN5 knockdown exhibited significantly enhanced cell migration and invasion (Fig. 2h, *P* < 0.001). More importantly, SLFN5-knockdown-mediated invasion enhancement was further confirmed by in vivo chick embryo CAM invasion assays,^39–43^ and both the percentage of invasive cells and the invasion depth increased following SLFN5 knockdown (Fig. 2i, *P* < 0.001). Next, we performed rescue experiments using human SLFN5, as mouse Slfn5 has only 59% homology to humans. The results showed that SLFN5 transfection rescued SLFN5 knockdown, resulting in E-cadherin and vimentin changes (Fig. 2j, *P* < 0.01). Together, these findings indicate that SLFN5 knockdown can both morphologically and functionally induce EMT in epithelial-like BRCA cells.

### Overexpression of SLFN5 results in MET in mesenchymal-like BRCA cells

**Fig. 3:**
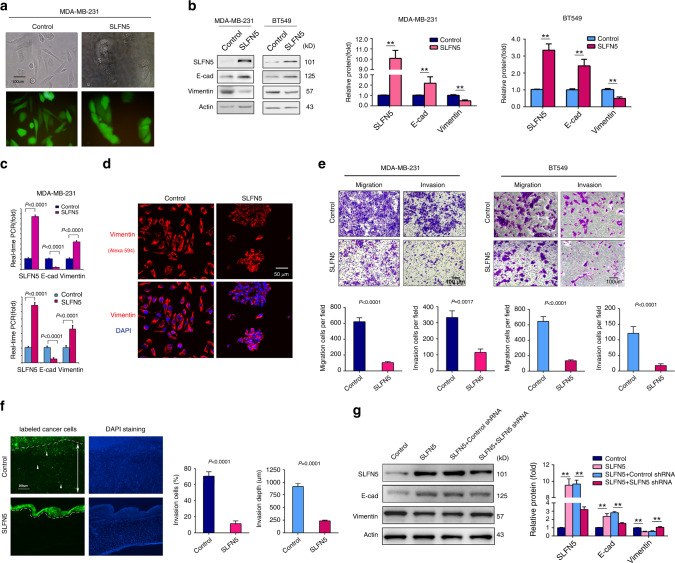
Overexpression of SLFN5 results in MET in mesenchymal-like BRCA cells and suppresses cell invasion in vitro and in vivo. **a** Contrast-phase and fluorescent images of MDA-MB-231 cell lines stably transfected with a lentiviral control or the lentivirus carrying SLFN5. **b** WB analyses of SLFN5, E-cad and vimentin protein expression in MDA-MB-231 and BT549 cell lines stably transfected with a lentiviral control or the lentivirus carrying SLFN5. Protein band intensity is quantified and presented as the mean ± SD of three independent experiments. **P < 0.01. **c** Real-time PCR analyses of SLFN5, E-cad and vimentin protein expression in MDA-MB-231 and BT549 cell lines stably transfected with a lentiviral control or the lentivirus carrying SLFN5. **d** Immunofluorescence analysis of vimentin protein in MDA-MB-231 cell lines stably transfected with a lentiviral control or the lentivirus carrying SLFN5. Staining was performed with a vimentin antibody, which was followed by labelling with an Alexa Fluor 594-conjugated secondary antibody. The results were observed with laser confocal fluorescence microscope. DAPI staining revealed the nuclei. **e** Migration and invasion of MDA-MB-231 or BT549 cell lines stably transfected with a control or SLFN5 overexpression vector. Scale bar: 50 μm. **f** Chick CAM invasion analysis of MDA-MB-231 cell lines transfected with a control or SLFN5 overexpression vector. The white dotted lines indicate the position of the CAM. The white arrowheads indicate cells invading into the chick embryos. The double-headed arrow indicates invasion depth in the chick embryo. **g** Rescue experiments were performed using human SLFN5 shRNA viral particles or vector control viral particles. The results show that SLFN5 shRNA transfection partially rescued SLFN5 overexpression-induced E-cadherin and vimentin changes. Protein band intensity is quantified and is presented as the mean ± SD of three independent experiments. **P < 0.01.

To further investigate the role of SLFN5 in EMT in BRCA cells, we overexpressed SLFN5 in MDA-MB-231 and BT549 cells. SLFN5 overexpression apparently triggered MET, as evidenced by morphological changes from a mesenchymal-like morphology to a cobblestone epithelial morphology (Fig. 3a). Correspondingly, SLFN5 overexpression resulted in a decrease in vimentin, and an increase in E-cad in both MDA-MB-231 and BT549 cells (Fig. 3b, c, *P* < 0.01 or *P* < 0.001). Furthermore, the SLFN5-mediated decrease in vimentin was also confirmed by IF staining (Fig. 3d). In addition, both the in vitro Transwell migration/invasion and the in vivo chick embryo CAM invasion assays showed that SLFN5 overexpression significantly suppressed migration and invasion in BRCA cells (Fig. 3e, f). Next, rescue experiments were performed by transfection of SLFN5 shRNA into SLFN5-overexpressing stable cell lines, and the results showed that SLFN5 shRNA transfection significantly attenuated the SLFN5 overexpression effect on e-cadherin and vimentin expressions (Fig. 3g, *P* < 0.01). Together, these results indicate that SLFN5 can both morphologically and functionally trigger MET in mesenchymal-like BRCA cells.

### ZEB1 is a mediator of SLFN5-regulated EMT

**Fig. 4:**
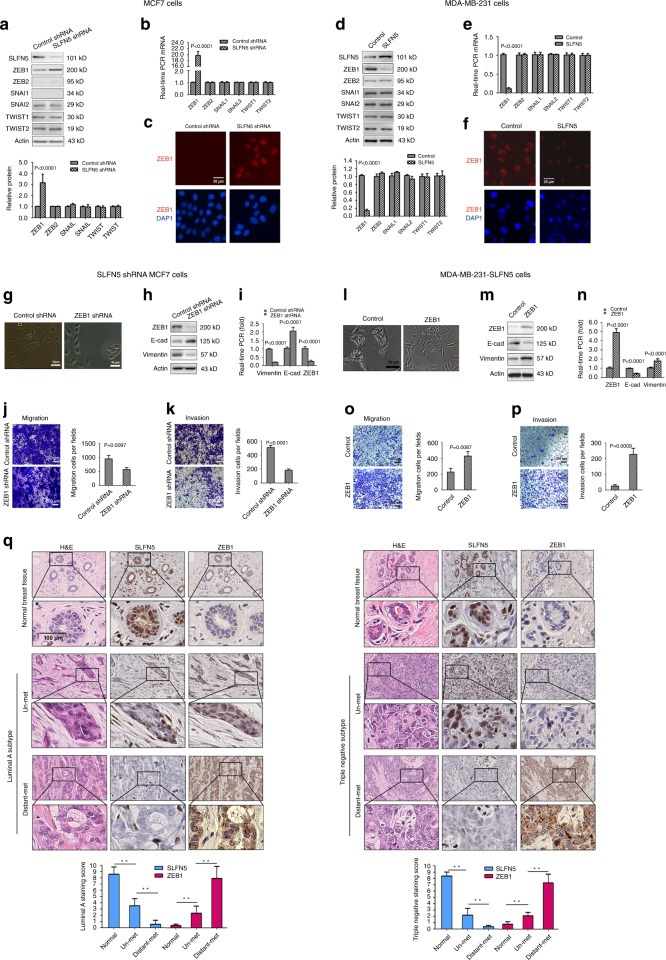
ZEB1 is a mediator of SLFN5-regulated EMT. **a**, **b** WB and real-time PCR analyses of the protein and mRNA expression of EMT transcription factors in MCF7 cells stably transfected with control shRNA or SLFN5 shRNA. **c** Immunofluorescence staining of ZEB1 enhancement by SLFN5 shRNA transfection. **d**, **e** WB and real-time PCR analyses of the protein and mRNA expression of EMT transcription factors in MDA-MB-231 cells stably transfected with control or SLFN5 overexpression vector. **f** Immunofluorescence staining of ZEB1 downregulation following transfection with a SLFN5 overexpression vector. **g** Morphological changes in MCF7 cells stably transfected with SLFN5 shRNA combined with either control shRNA or ZEB1 shRNA. **h**, **i** WB and real-time PCR analyses of the protein and mRNA expression of ZEB1, E-cad and vimentin in MCF7 cell lines stably transfected with SLFN5 shRNA combined with control shRNA or with ZEB1 shRNA. **j**, **k** Migration and invasion determination of MCF7 cells stably transfected with SLFN5 shRNA combined with either control shRNA or ZEB1 shRNA. **l** Morphological changes in MDA-MB-231 cells stably transfected with SLFN5 combined with either control or ZEB1 overexpression vector. **m**, **n** WB and real-time PCR analyses of the protein and mRNA expression of ZEB1, E-cad and vimentin in MDA-MB-231 cells stably transfected with SLFN5 combined with either control or ZEB1 overexpression vector. **o**, **p** Migration and invasion determination of MDA-MB-231 cells stably transfected with SLFN5 combined with either control or ZEB1 overexpression vector. **q** Immunohistochemistry analyses of SLFN5 and ZEB1 expression in clinical BRCA luminal A and triple-negative subtypes. Staining scores of SLFN5 and ZEB1 were calculated based on the percentage of positive cells and staining intensity (mean ± SD, *n* = 5). SLFN5 and ZEB1 expression exhibited opposite trends. H&E staining was used to identify pathological features of cancer. **P < 0.01.

It is well known that SNAI1, SNAI2, ZEB1, ZEB2, TWIST1 and TWIST2 are key transcription factors involved in EMT in various types of cancers, and they contribute to enhanced tumour cell migration, invasion and metastasis capacities. These transcription factors were examined in SLFN5-knockdown MCF7 cells and SLFN5-overexpressing MDA-MB-231cells. SLFN5 knockdown significantly increased ZEB1 expression, but SLFN5 overexpression significantly decreased ZEB1 expression, at both the mRNA and protein levels (Fig. 4a–c, d–f, both *P* < 0.001), whereas ZEB2, SNAIl, SNAI2, TWIST1 and TWIST2 showed no significant changes (Fig. 4a, b, d, e). Similar results were obtained in BT549 cells overexpressed with SLFN5 (Supplementary Fig. S4). Our findings here are consistent with a previous finding that ZEB1 was found to be mainly overexpressed in basal-like breast cancer by Katsura et al.^44^

To further determine whether ZEB1 is a mediator of SLFN5-regulated EMT, knockdown of ZEB1 was performed in SLFN5-silencing MCF7 cells. ZEB1 knockdown apparently reversed the phenotype induced by SLFN5 silencing, as evidenced by reduced numbers of mesenchymal-like cells (Fig. 4g), increased E-cad expression and decreased vimentin expression (Fig. 4h, i, *P* < 0.001). Moreover, ZEB1 knockdown significantly impaired the migration and invasion capacities of SLFN5-silencing MCF7 cells (Fig. 4j, k, *P* = 0.0097 or *P* < 0.001). Furthermore, forced ZEB1 expression in SLFN5-overexpressing MDA-MB-231 cells significantly reversed SLFN5-mediated MET, decreased E-cad expression, increased vimentin expression and enhanced cell migration and invasion (Fig. 4l–p, *P* < 0.001, *P* = 0.0087 or *P* = 0.0009).

In addition, to further investigate their relationship, we detected SLFN5 and ZEB1 expression in clinical BRCA samples using immunostaining method, and found that in each subtype of BRCA, they showed an opposite trend. That is, ZEB1 levels increased in cancer tissues without metastasis compared with normal tissues, and they further increased in localised BRCA tissues with distant metastasis, contrary to the SLFN5 expression pattern in BRCA (Fig. 4q, luminal A and triple negative; Supplementary Fig. S5, luminal B and HER2^+^).

### SLFN5 inhibits *ZEB1* gene transcription by binding to specific sites within the *ZEB1* promoter region

**Fig. 5:**
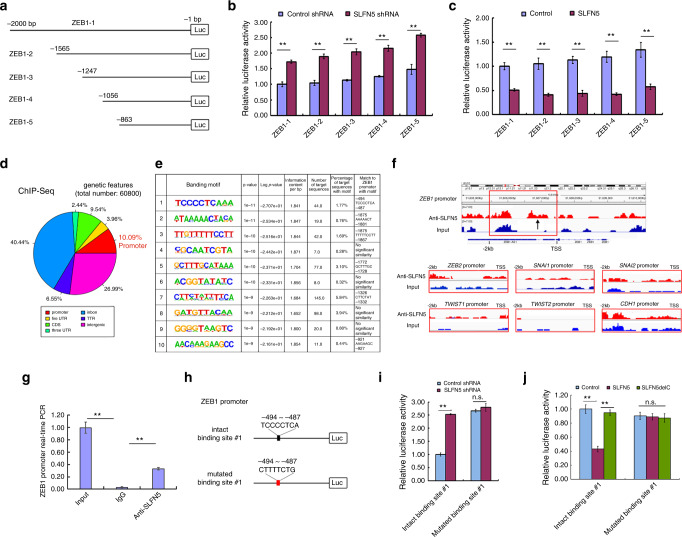
ChIP-Seq and luciferase activity analyses of SLFN5 inhibition of *ZEB1* transcription by binding to specific sites within the *ZEB1* promoter region. **a** Schematic diagram of luciferase reporter gene constructs with the *ZEB1* promoter (TSS upstream from –1 to –2000 bp) or the indicated length promoters. **b**, **c** Relative luciferase activity analyses of the *ZEB1* promoter with the indicated length in MCF7 cells stably transfected with control shRNA or SLFN5 shRNA (**b**), or in MDA-MB-231 cells stably transfected with lentiviral control or lentivirus-overexpressing SLFN5 (**c**). **P < 0.01. **d** ChIP-Seq analysis of genetic features of chromosome fragments immunoprecipitated by SLFN5 antibody in MCF7 cells. **e** Putative SLFN5-binding motifs with the top-10 scores according to ChIP-Seq analysis. **f** ChIP-Seq data analysis of binding peaks of SLFN5 at promoters of *ZEB1*, *ZEB2*, *SNAI1*, *SNAI2*, *TWIST1*, *TWIST2* and *CDH1* (E-cadherin) in MCF7 cells with cell lysate input as a control. **g** Real-time PCR analyses of the *ZEB1* promoter in SLFN5 antibody-immunoprecipitated chromosome DNA. Cell lysate input was used as a positive control, and IgG immunoprecipitation was used as a negative control. **P < 0.01. **h** Schematic diagram of luciferase reporter gene constructs with *ZEB1* promoter mutants of the putative SLFN5-binding site #1. The black box indicates an intact wild-type binding site, and the red box indicates a mutated binding site. **i**, **j** Relative luciferase activity analyses of *ZEB1* promoter mutants of the putative SLFN5-binding site #1 in MCF7 cells stably transfected with control shRNA or SLFN5 shRNA (**i**), or in MDA-MB-231 cells stably transfected with lentiviral control, full-length wild-type SLFN5 expression vector or C-terminal deletion mutant (SLFN5delC) expression vector (**j**). **P < 0.01. n.s. indicates non-significant.

A previous study suggested that SLFN5 has a predicted nuclear localisation sequence in its C terminus,^45^ and our IF staining assays confirmed the nuclear localisation of SLFN5 (Fig. 2e), indicating that SLFN5 may have a potential regulatory function on gene transcription. To further determine whether SLFN5 could transcriptionally regulate ZEB1 expression, the activity of truncated *ZEB1* promoters (Fig. 5a) was tested in luciferase reporter assays. As shown in Fig. 5b, c, knockdown or overexpression of SLFN5 could induce or suppress luciferase activity, respectively (*P* < 0.01), suggesting that SLFN5 could serve as a transcriptional factor to direct *ZEB1* transcription. To determine the putative binding sites of SLFN5 on the *ZEB1* promoter, a ChIP-Seq assay was performed in MCF7 cells. A quantitative analysis-mapped SLFN5 reads was 10.09% in promoter and 62.92% in gene body locations, including intron, coding sequence (CDS), 5′ untranslated region (UTR), 3′UTR and transcriptional termination region (TTR) (Fig. 5d). Among the top ten putative SLFN5-binding motifs, six of them may reside in the *ZEB1* promoter region (from the transcriptional starting site (TSS) to –2000 bp) (Fig. 5e). Next, five out of the six putative binding sites, except the binding site #1 in the ZEB1 promoter, were excluded from further experiments because their deletions did not affect SLFN5-mediated *ZEB1* promoter activity (Fig. 5a–c).

According to the ChIP-Seq data, the SLFN5-binding peak was present in the *ZEB1* promoter (Fig. 5f), and this was further confirmed with real-time PCR (Fig. 5g, *P* < 0.01). However, marked binding peaks were not present in the promoters of *ZEB2*, *SNAI1*, *SNAI2*, *TWIST1*, *TWIST2* or E-cadherin (*CDH1*) (Fig. 5f). The microRNA (miR)-200 family is one of the major negative regulators of ZEB1 levels.^23^ However, no binding peak was present in the promoters of members of this family, including miR-200a, b, c and -141 (Supplementary Fig. S6). Furthermore, neither knockdown nor overexpression of SLFN5 influenced miR-200 family member levels, as assessed by real-time PCR analysis (Supplementary Fig. S7). Together, these exclude the influence of SLNF5 on ZEB1 transcription that is not due to an action on the miR-200 family.

To examine whether the putative binding site #1 is functionally involved in SLFN5-mediated *ZEB1* expression changes, we constructed *ZEB1* luciferase reporter vectors that carried either the intact wild-type promoter (5′-TCCCCTCA-3′) or a promoter that was mutated (5′-CTTTTCTG-3′) at binding site #1 (Fig. 5h). Luciferase assays demonstrated that SLFN5 was able to regulate luciferase activity in the reporter vector with intact wild-type binding site #1 (*P* < 0.01), but not in the vector with mutated binding site #1 (Fig. 5i, j), suggesting that SLFN5 can transcriptionally regulate *ZEB1* by binding to the motif (5′-TCCCCTCA-3′) in the *ZEB1* promoter. The C-terminal region of the SLFN5 structure contains some potential transcriptional regulation-related sequences or domains, such as a nuclear localisation signal sequence and a motif homologous to the RNA/DNA helicase superfamily.^45^ Compared with full-length wild-type SLFN5, the C-terminal deletion mutant (SLFN5delC; C-terminal 335AA was deleted) was unable to regulate *ZEB1* promoter activity (Fig. 5j), suggesting that SLFN5 depends on its C-terminal transcriptional regulatory sequences to inhibit ZEB1 transcription.

## Discussion

Human SLFN5 expression is correlated with tumour cell invasion and metastasis in several malignancies. However, its precise implication in BRCA has not been defined. In the present study, we demonstrate that human SLFN5 expression shows a gradually decreasing trend throughout BRCA progression in patients. SLFN5 is expressed in unmetastasised epithelial-like BRCA cells, but poorly expressed in metastasised mesenchymal-like BRCA cells. Morphologically and physiologically, knockdown of endogenous SLFN5 can induce EMT, whereas overexpression of exogenous SLFN5 can trigger MET in BRCA cells. Mechanistically, we demonstrate that SLFN5 can directly bind to the binding motif (5′-TCCCCTCA-3′) in the promoter region of *ZEB1* to transcriptionally inhibit *ZEB1* expression.

The process of tumour metastasis consists of multiple steps, i.e., EMT, invasion of the extracellular matrix and basement, MET and colonisation.^12–15^ EMT is a key initial step in mobilising some epithelial cancers to metastasise. Invasion of the extracellular matrix is another important step for cancer metastasis to distant sites.^46,47^ In our previous studies, we demonstrated that SLFN5 can impress cancer cell invasive capacity by inhibiting MT1-MMP expression. Our present findings showed that SLFN5 can maintain or restore epithelial morphology, and inhibit the invasion and metastasis of cancer cells. Together, our findings suggest that SLFN5 plays inhibitory roles in cancer cell metastasis. However, whether SLFN5 is implicated in other steps in cancer metastasis is unknown.

The function of SLFN5 protein (as well as other members of the family) is still largely unknown. SLFN5 nuclear localisation tends to support some transcriptional regulatory action, but this has not been fully established. Work by other authors has reported an SLFN5 co-repressor action with STAT1 in IFN-mediated responses, by promoting malignant progression in glioblastoma,^10^ but a direct transcriptional role has not been yet reported. In our current study, we provide some evidence for such a role. First, both our IHC staining of the BRCA clinical samples and the IF staining in the BRCA cells showed marked staining of SLFN5 in the nucleus. Second, our knockdown and overexpression data demonstrated that SLFN5 can regulate ZEB1 transcription. Third, our ChIP-Seq data further suggested that SLFN5 may function as a transcriptional factor because 10% of SLFN5-specific reads are located in promoter regions. We also demonstrated that binding motif #1 (5′-TCCCCTCA-3′) is a functional motif for SLFN5-mediated ZEB1 expression regulation by both ChIP-Seq and luciferase reporter assays. Fourth, human SLFN5 could have two special motifs in its long C terminus: a nuclear localisation signal and a motif homologous to the RNA/DNA helicase superfamily;^45^ we constructed the C-terminal deletion mutant (SLFN5delC) and found that it was deficient in influencing ZEB1 promoter activity, further suggesting that SLFN5 depends on its C-terminal regulatory sequences to perform ZEB1 transcriptional regulation. However, the function of SLFN5 protein remains largely unknown. SLFN5 may perform other functions via binding to introns, coding sequences, 5′UTRs or 3′UTRs.

ZEB1 is aberrantly expressed in human BRCA and promotes BRCA metastasis.^33,34^ It has been identified as the key transcription factor for cell plasticity, local invasion and distant metastasis in mouse pancreatic cancer,^35^ since depletion of Snai1 or Twist1 in this model is unable to affect metastatic processes. In our present study, SLFN5 solely affected the expression of ZEB1 but not other factors: SNAI1, SNAI2, TWIST1, TWIST2 and ZEB2. As reported, many factors can transcriptionally regulate ZEB1 expression. For instance, Ets1 induces *ZEB1* transcription in BRCA by binding to 5′-GGAT-3′ in the *ZEB1* promoter; MEF2 activates *ZEB1* transcription in colorectal cancer by binding to sequences rich in A/T, such as 5′-TAAATTTAT-3′, 5′-ATAATTTTAA-3′ and 5′-TTAATTTATA-3′ within the *ZEB1* promoter; ISL1 induces *ZEB1* transcription in gastric cancer by binding to TAAT in the ZEB1 promoter; Notch3 activates *ZEB1* transcription by binding to the *ZEB1* promoter, possibly through the Notch family-related 5′-RTGGGAA-3′ response element. In our current study, we demonstrate that SLFN5 suppresses *ZEB1* transcription by binding to 5′-TCCCCTCA-3′; this motif cannot be found in the promoter regions of *SNAI1*, *SNAI2*, *TWIST1*, *TWIST2* and *ZEB2*. Regarding the probable mediation of the miRNA-200 family between SLFN5 and ZEB1, it was ruled out since miRNA-200 family levels were not influenced by knockdown or overexpression of SLFN5, suggesting that SLFN5 directly regulates ZEB1 transcription.

Taken together, in this study, we found that human endogenous and exogenous SLFN5 can maintain or restore breast cancer cells with epithelial morphology by transcriptional suppression of ZEB1 expression. Increasing evidence indicates that ZEB1 exerts functions in addition to regulating morphology.^48^ More than 2000 downstream genes are regulated by ZEB1, suggesting that ZEB1 may be involved in a central switch that controls key cellular functions and states.^32,49^ In this case, SLFN5 may be more deeply involved in cancer progression via ZEB1 mediation, which needs to be studied further. In addition, precise structures in SLFN5 that mediate its regulation of ZEB1 transcription need to be further investigated. Our findings indicate that human SLFN5 may be a potential novel target for BRCA therapy. Recent studies demonstrate that the intermediate epithelial/mesenchymal state of cancer cells may allow them to re-epithelialise at distant metastatic sites and colonise.^14,15,50^ Therefore, if a gene, such as SLFN5, is considered for future cancer treatment, it needs to be efficiently and stably transfected and expressed in all target cancer cells to avoid the intermediate epithelial/mesenchymal states.

## Supplementary information

Supplementary Information

## Data Availability

Supplementary data are available for this paper.
