# Supplementary material for: Human Schlafen 5 regulates reversible epithelial and mesenchymal transitions in breast cancer by suppression of ZEB1 transcription
Source: Br J Cancer. 2020 Jun 3;123(4):633–43. doi: 10.1038/s41416-020-0873-z (PMC7435190; doi:10.1038/s41416-020-0873-z)
Supplement: Supplementary file 1 — Supplementary Information [file 41416_2020_873_MOESM1_ESM.docx]

**Supplementary tables and figures**

**Table S1. Primers used in the present study.**

| Homo gene or promoter | forward and reverse primers | product (bp) |
| --- | --- | --- |
| SLFN5 | 5'-AAGACAGGCTTCTAACAGCAATGAGG-3'  5'-CATCCGACGCATCACCGATCTG-3' | 93 |
| E-cadherin | 5'-CTGAGAACGAGGCTAACG-3'  5'-GTCCACCATCATCATTCAATAT-3' | 111 |
| ZO-1 | 5'-GGCGGATGGTGCTACAAGTGATG-3'  5'-GCCTTCTGTGTCTGTGTCTTCATAGTC-3' | 139 |
| vimentin | 5'-TCGTGAATACCAAGACCTGCTCAATG-3'  5'-AATCCTGCTCTCCTCGCCTTCC-3' | 94 |
| ZEB1 | 5'-TGGCGGTAGATGGTAATGTAATAAGGC-3'  5'-GCTAGGCTGCTCAAGACTGTAGTTG-3' | 197 |
| ZEB2 | 5'-GTGCAAGAGGCGCAAACAAGC-3'  5'-GAATCTCGTTGTTGTGCCAGG-3' | 275 |
| SNAIL1 | 5'-CAGAGTTTACCTTCCAGCAGC-3'  5'-GACTCTCCTGGAGCCGAAGG-3' | 168 |
| SNAIL2 | 5'-GCCAAACTACAGCGAACTGG-3'  5'-GCCATTGGGTAGCTGGGCGTG-3' | 169 |
| TWIST1 | 5'-GGAAGATCATCCCCACGCTG-3'  5'-GCCATCTTGGAGTCCAGCTC-3' | 124 |
| TWIST2 | 5'-CGCTACAGCAAGAAGTCGAG-3'  5'-CTTGCTCAGCTTGTCAGAGG-3' | 210 |
| β-actin | 5ˊ-TACCTCATGAAGATCCTCACC-3ˊ  5ˊ-TTTCGTGGATGCCACAGGAC-3ˊ | 268 |
| hsa-miR-200a | 5'-ACACTCCAGCTGGGTAACACTGTCTGGTAACG-3'  5’ CTCAACTGGTGTCGTGGA-3' | 72 |
| hsa-miR-200b | 5'-ACACTCCAGCTGGGTAATACTGCCTGGTAATG-3'  5’-CTCAACTGGTGTCGTGGA-3' | 72 |
| hsa-miR-200c | 5'-ACACTCCAGCTGGGTAATACTGCCGGGTAATG-3'  5'-CTCAACTGGTGTCGTGGA-3' | 73 |
| hsa-miR-141 | 5'-ACACTCCAGCTGGGTAACACTGTCTGGTAAAG-3'  5'-CTCAACTGGTGTCGTGGA-3' | 72 |
| U6 | 5'-CTCGCTTCGGCAGCACA-3'  5'-AACGCTTCACGAATTTGCGT-3' | 94 |
| ZEB1 promoter fragment | 5'-CCTGGAAGGGAAGGGAAGGG-3'  5'-ACCCTGTGCCCTCGGAGCTGC-3' | 222 |
| mutSLFN5DelC | 5'-ccgctcgaggccaccATGAGTCTTAGGATTGATGTGGATAC-3'  5'-cggggtaccCTATATCACGAGGGACTGTAACAGGGCTTCC-3' |  |

**
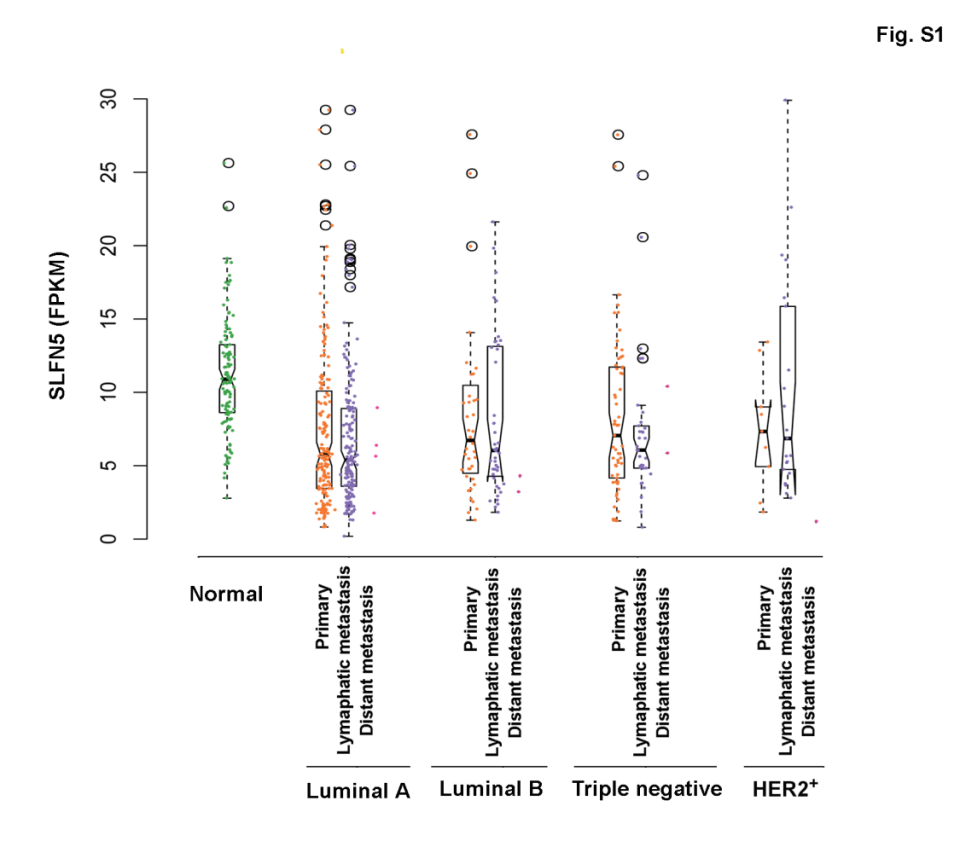
**

**Fig S1. Analyses of human SLFN5 expressions in BRCA subtypes using TCGA data.**

**
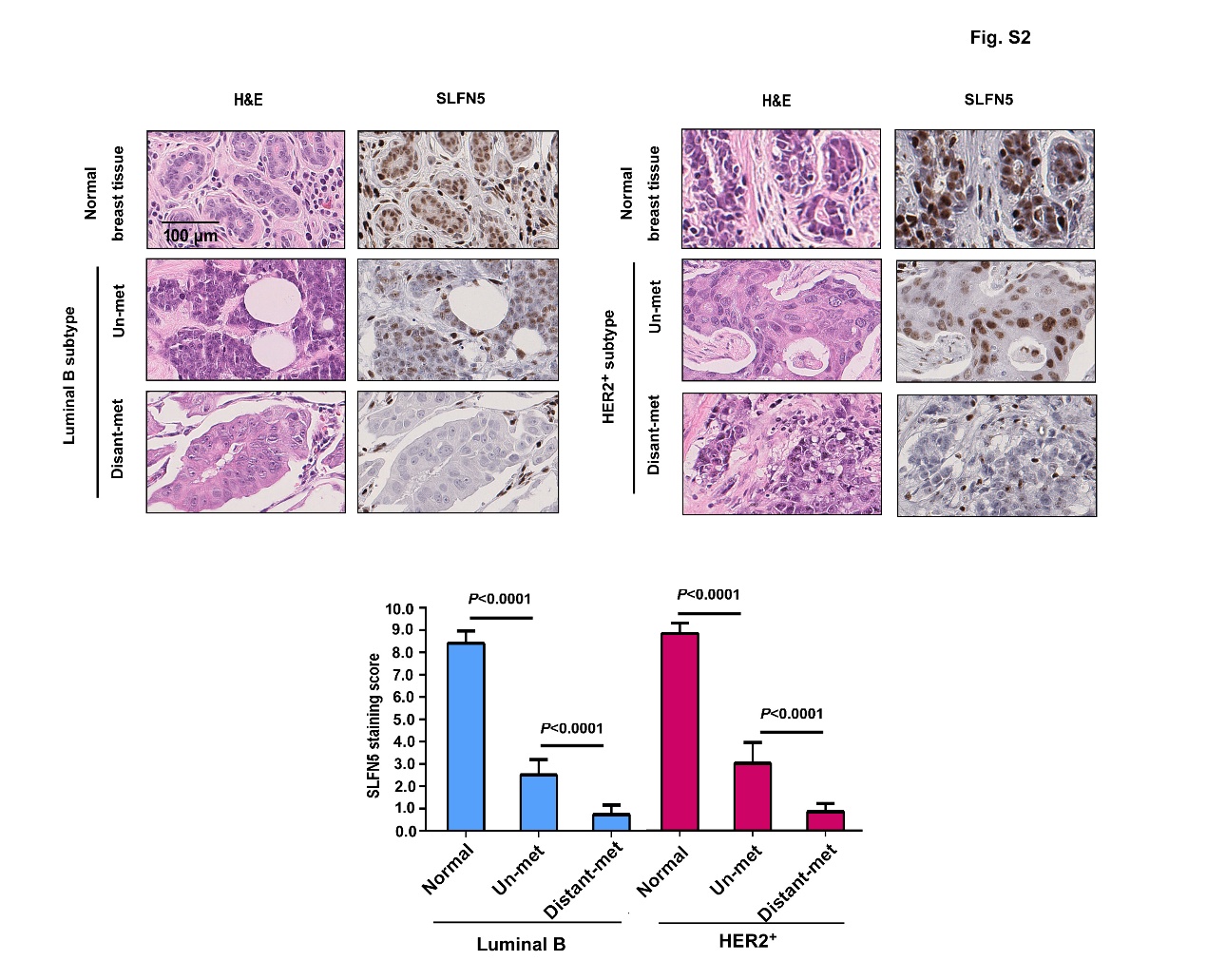
**

**Fig. S2.** Immunohistochemistry analysis of SLFN5 protein levels in BRCA subtype Luminal B and HER2^+^. SLFN5 expression was significantly less in BRCA tumor tissues of these two subtypes than that in normal tissues, and further decreased in local primary BRCAs with distant metastasis (Distant-met) compared with those without metastasis (Un-met). SLFN5 expression scores were calculated based on the percentage of positive cells and the staining intensity, with a total score of 10 points. H&E staining was performed to identify cancer pathological features. Scale bar: 100 μm.

**
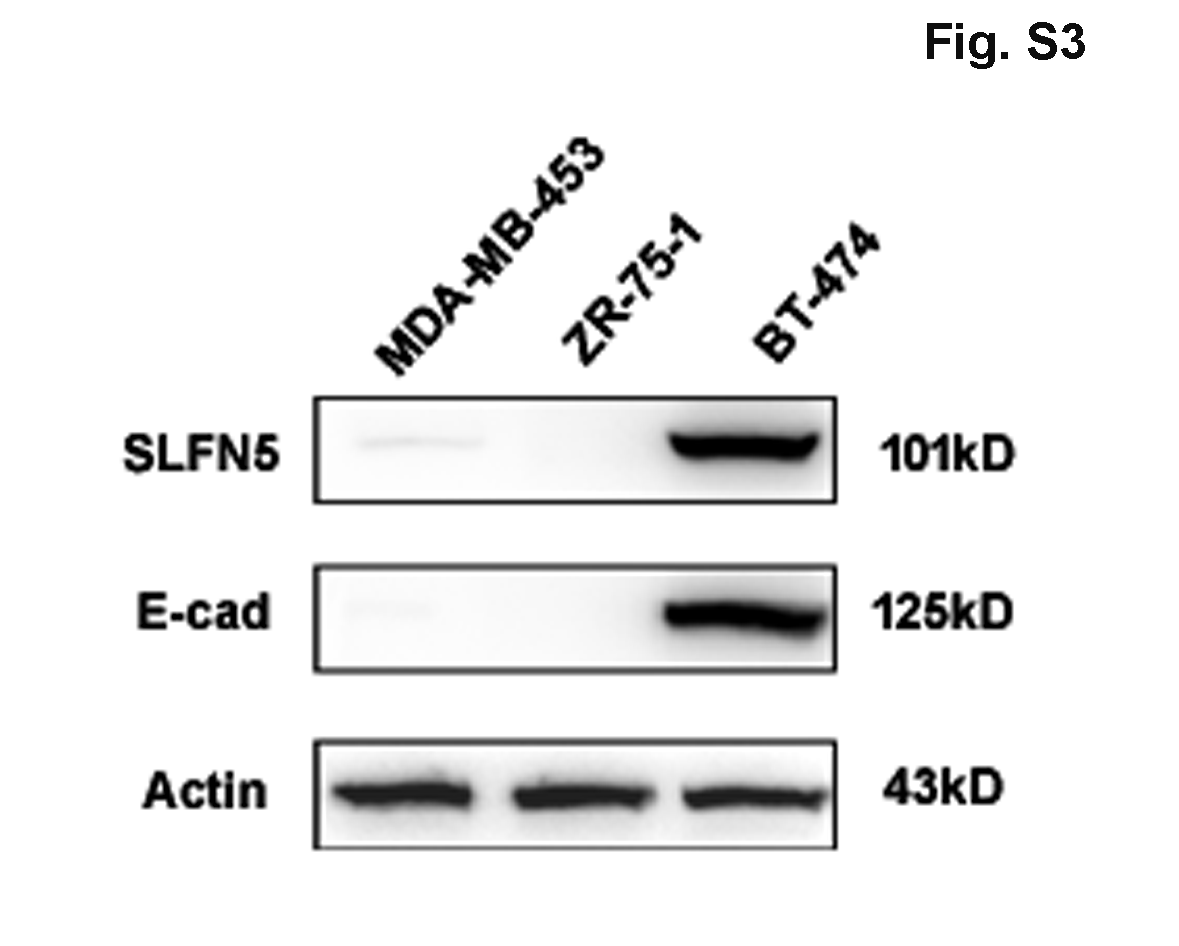
**

**Fig. S3.** WB assays of SLFN5 expressions in Luminal B and HER2 subtype cell lines. Results showed high expressions of SLFN5 in low metastasized BRCA cell lines, but low expressions of SLFN5 in high metastasized BRCA cell lines.


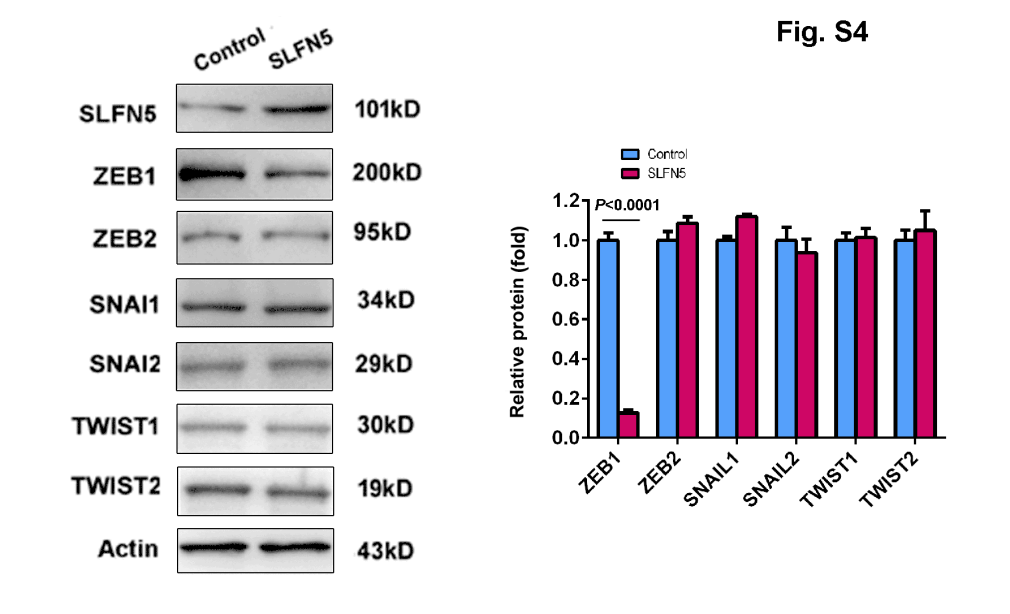


**Fig. S4.** WB assays of transcription factor expressions in SLFN5 knocked down BT549 cells.

**
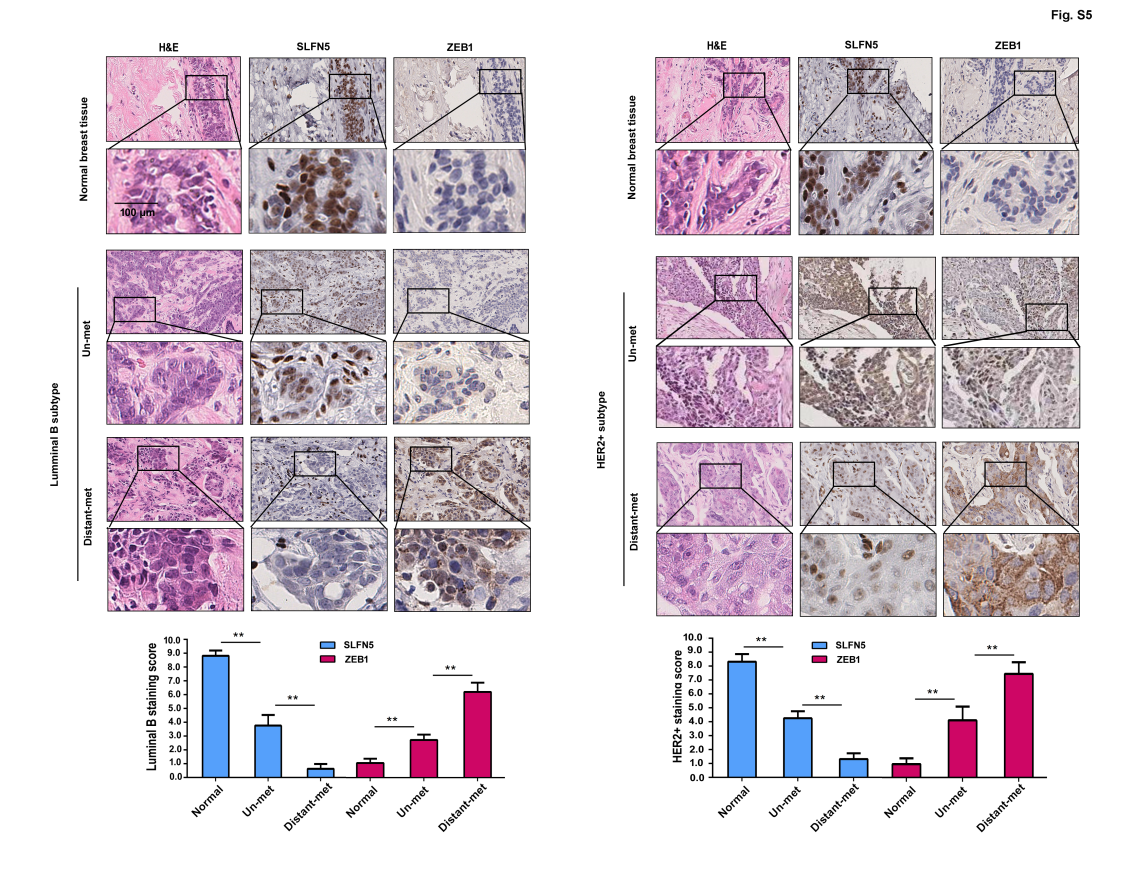
**

**Fig. S5.** Immunohistochemistry analysis of co-localization of SLFN5 and ZEB1 in clinical BRCA Luminal B and HER2^+^ subtypes. Results showed that SLFN5 and ZEB1 expression patterns present an opposite trend, that is, ZEB1 increased in cancer tissues compared with normal tissues, and further increased in localized BRCA tissues with distant metastasis. ** *P* < 0.01. Scale bar: 100 μm.

**
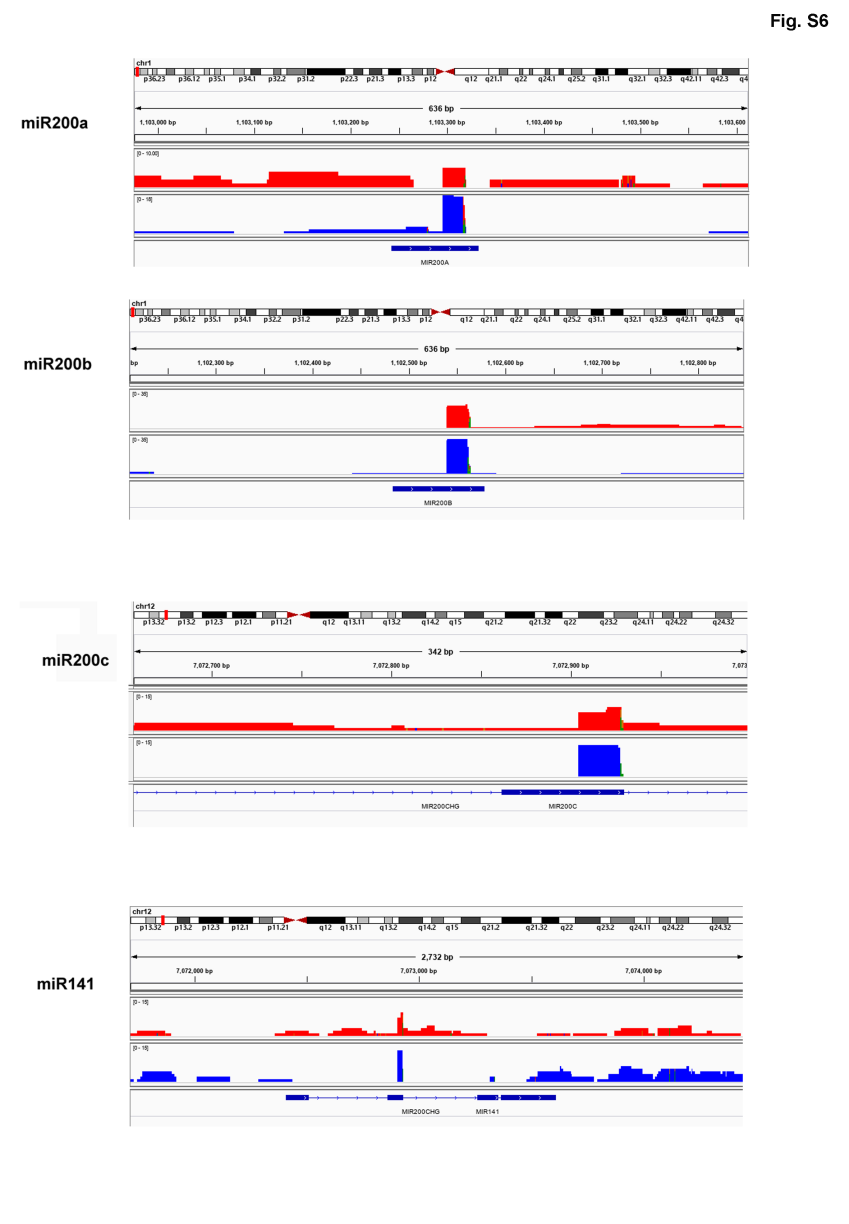
**

**Fig. S6.** ChIP-seq analysis of binding peaks of SLFN5 on the promoter regions of miRNA 200 family members. No binding peak was found on the promoters of miRNA 200 family, including miR-200a, b, c and -141.

**
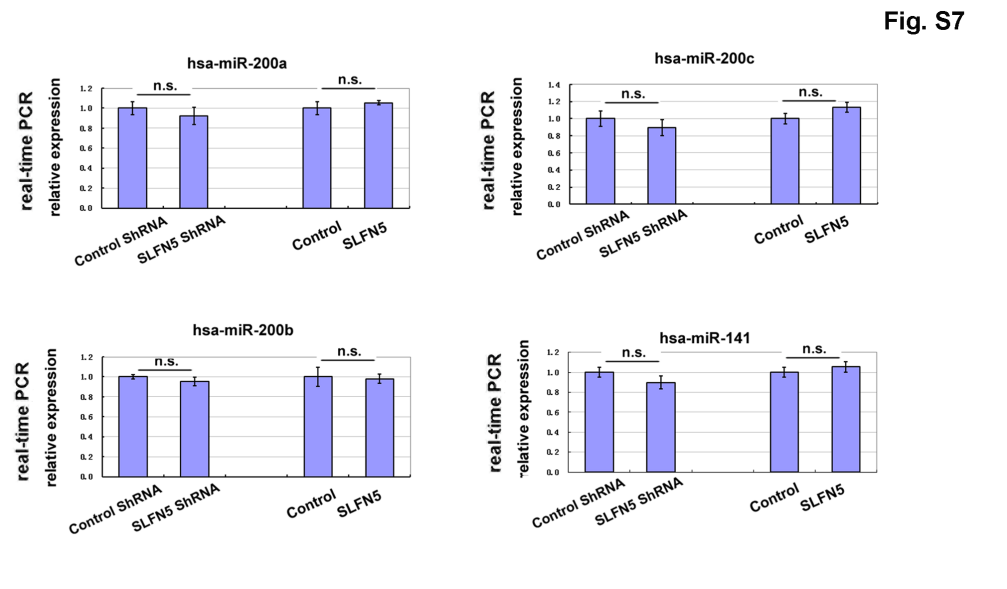
**

**Fig. S7.** Real-time PCR analyses of miR-200 family levels in MCF7 cells stably transfected with control shRNA or SLFN5 shRNA, or in MDA-MB-231 cells stably transfected with control or SLFN5. Neither knockdown nor overexpression of SLFN5 influenced miR-200 family levels. n.s., non-significant.
